# Tardy Eruption of the Teeth

**Published:** 1880-07

**Authors:** Scribulus Scifendi


### TARDY ERUPTION OF THE TEETH.

BY SCRIBULUS SCIFENDI.

Retarded eruption of one or more of the permanent teeth is frequently noticed, in my mouth is exhibited an instance. In 1831, my left superior incipient cuspid was not shed, while all my other permanent teeth were in position and in good condition, the incipient tooth was unsightly and looking unprofessional, I had it extracted and wore an artificial tooth until 1870 and '71, when I inserted a full upper set. For three or four years after I experienced occasionally a sharp pain about the position of the left central incisor, would occasionally remove the plate. In May,

1878, in passing my tongue over the ridge, felt a roughness, and on closer examination I discovered nearly an entire tooth, but in a decayed condition, transversely with the jaw, or as the sailor would express it "broad side." It has been very backward in coming forward, for it should have made its advent over 60 years ago.

What changes have passed over its possessor during its long and solitary seclusion within the integuments, for he has seen, (and known,) Empires to be formed and toppel over; Kings crowned and uncrowned, nation arising against nation, filling thousands of hearts with grief, and drenching the land in blood, a golden State made redolant with the hum of progressive humanity; his Country adding almost empires to her borders; the old union nearly rent asunder; slavery abolished; (a blessing in disguise); all this and more has passed old *Cuspidatus*, while you have been snugly ensconced, "unharmed by the war of elements, the wreck of matter and the crash of"—well of many of your compeers in usefulness—but what good have you done by coming?

On further reading of the proceedings of the Ohio Dental Association, I observed Dr. Ambler's report of retarded dentition. The tooth in the lady's case was like mine, the left superior cuspid, came when she was 52 years of age, mine when I was about 74. My unpleasant reverse of future is explained, by my not having "cut my eye teeth."

A few years since I had a young lady patient who at 20 years old, had not "*cut*" her superior permanent incisors, cuspids or bicuspids and the bicuspids of the lower set. As there was no evidence of their appearance, and she said it was thought an M. D. had by mistake, (ignorance) extracted the permanent, I inserted a partial denture in each jaw. In about two years she complained of the upper plate being loose, on examining the mouth I found the cutting edge of the right incisor just through the gum—adapted the plate to that, all was well for a year—upon her calling again found all the absentees reporting themselves, and the plates were dispensed with.

In the mouth of another patient I found *six* supernumary *bicuspids*, all well developed bicuspids—two on the palatine side of each of the regular teeth, and one near each of the wisdom teeth. I took an impression of the mouth and sent a cast, I think, to the Baltimore College.

I had a superior dens sapintia I extracted some years since, exhibiting *five* well developed roots. My patient was a large-framed negro, with the most *capacious oral cavity* I recollect of seeing, the superior wisdom teeth were as easily reached as are ordinary bicuspids.

The foregoing may be regarded as *incidents in* practice—the *accidents of* practice have been occasional, if not oftener—as one instance, a lady called to have the second sup. molar extracted. I applied the *turnkey* (this was long years ago) with every confidence of success, and of a truth I was not disappointed, for I removed the molar and the *first* and *third*, and the alveolus with the roof of the arch. Considering that extract worthy of a place in the volume of "Beautiful American Extracts," I have since discarded the *key* instrument, fearing I might overshadow that splendid per formance. I will incidently remark, that on applying force to the instrument my patient caught my right wrist with both her hands —a fact which might have had some influence in giving the key an undesired direction—that was my impression, and some year or two after the untoward event, my patient admitted having embraced me with more energy than was required.

It is possible, Dr. Taft, you may "keep a corner in the thing you use (the *Register*) for other's uses," if such is the case and my jottings are suitable, they are at your service.

